# Network-based pharmacology to predict the mechanism of qianghuo erhuang decoction in the treatment of rheumatoid arthritis

**DOI:** 10.4314/ahs.v24i4.37

**Published:** 2024-12

**Authors:** Xuemeng Chen, Qinghua Zou, Bing Zhong, Yong Wang

**Affiliations:** Department of Traditional Chinese Medicine, Southwest Hospital, the Third Military Medical University (Army Medical University), Chongqing (400038), China

**Keywords:** Computer communication networks, Rheumatoid arthritis, Target

## Abstract

**Background:**

To investigate the mechanisms of Qianghuo Erhuang Decoction (QED) in the treatment of rheumatoid arthritis (RA), we performed compounds, targets prediction and network analysis using a network pharmacology method.

**Methodology:**

We collected active ingredients and targets of QED according to the database of Traditional Chinese Medicine System Pharmacology Database and Analysis Platform (TCMSP) and selected therapeutic targets on RA. “drug-ingredient-target” network was made for the intersecting genes. The STRING database was used for constructing a protein-protein interaction network (PPI) for the intersection genes, and R version 4.1.2 software was used for Gene Ontology (GO) and Kyoto Encyclopedia of Genes and Genomes (KEGG) enrichment analysis.

**Results:**

We found that there were 141 main active ingredients in QED, of which the main core active ingredients were: β-sitosterol, stigmasterol, baicalein, wogonin, kaempferol, etc., involving 166 RA genes. GO enrichment analysis results showed that QED involved 2229 biological processes, 78 cell components and 212 molecular functions. QED might interfere and treat RA through lipid and atherosclerosis, cancer pathways, PI3K-Akt, AGE-RAGE, IL-17, TNF, as well as HIF-1 signaling pathways.

**Conclusions:**

QED may treat RA by regulating inflammation-related signaling pathways, angiogenesis signaling pathways, and reducing the expression of inflammatory factors.

## Introduction

Rheumatoid arthritis (RA) is a chronic autoimmune disease characterized by joint swelling, synovial inflammation and cartilage destruction^1^. The precise pathogenesis of RA has not been completely elucidated. However, it was reported that infiltration of a large number of CD4+ T lymphocytes and hyperplasia of synoviocytes in the synovial membrane had triggered and exacerbated synovitis in RA1. Currently, non-steroidal anti-inflammatory drugs, disease-modifying anti-rheumatic drugs, glucocorticoids and biologic drugs are usually recommended for RA treatment. However, these drugs are associated with varying degrees of side effects including gastrointestinal disorders, immunodeficiency, infection and humoral disturbances, strongly suggesting the need to explore new therapeutic agents with low toxicity and high efficacy^2^,^3^. Complementary therapies based on Chinese medicines can be recommended as an attractive alternative^4^.

In Traditional Chinese medicine (TCM), RA is a kind of arthromyodynia (BI syndrome in Mandarin), and its symptoms are analogous to “Li-jie-bing”, “Feng-shi”, and “He-xi-feng”, as described in the Huangdi's Internal Classic and Plain Questions^5^. Chinese clinicians have demonstrated damp-heat impeding syndrome and cold-damp impeding syndrome as the most common RA patterns in patients^6^. Qianghuo Erhuang Decoction (QED), derived from ancient classical recipe Qianghuo Shengshi Decoction of Piwei Lun (Treatise on Spleen and Stomach) written by Li Dong Yuan, was composed of Qianghuo (Notopterygium incisum), Duhuo (Heracleum Linnaeus L.), Qinjiao (Gentiana macrophylla pall.), Yi Yiren (Coix lacryma-jobi L.), Ren Dongteng (Lonicerae japonica Tunb), Huangqin (Scutellaria baicalensis Georgi), Jianghuang (Curcuma longa L.), Ezhu (Curcuma zadoaria (Christm) Rosc.), Chuanxiong (Ligusticum chuanxiong Hort.), Gancao (Glycyrrhiza uralensis Fisch), which were combined in a ratio of 20:15:20:30:30:20:10:15:15:5. Treatment with this new traditional drug formulation could eliminate dampness and heat, activate blood circulation and relieve numbness, and therefore, could be more effective against rheumatism and heat-induced numbness^7^. Modern pharmaceutical studies indicated that the ingredients were compatible with each other, and played an anti-inflammatory, analgesic and immune-regulatory role^8^,^9^. Our study showed that the high-dose QED had a therapeutic effect against adjuvant arthritis and regulated the related cytokine levels in serum, which might be mediated via restoration of the imbalance in CD4^+^ T lymphocyte subsets, and the multi-target mechanisms of the herbs still need to be further clarified^7^.

The network pharmacology is a theory of system biology that can be used for drug target and mechanism prediction research. It provides a deeper insight or scientific evidence for TCM knowledge and helps us elucidate action mechanisms at a biological molecular level^10^,^11^. Therefore, based on the concept of network pharmacology, the bioinformatics method was used to predict the specific molecular mechanism of QED on RA, and provided a basis for clinical application.

## Materials and methods

### Drug active ingredients and active ingredient targets

Using the Traditional Chinese Medicine System Pharmacology Database and Analysis Platform (TCMSP) database (http://lsp.nwu.edu.cn/tcmsp.php) to search each Chinese medicine in QED, by enter the following keywords one by one: 1. Qianghuo, 2. Duhuo, 3. Qinjiao, 4. Yi Yiren, 5. Ren Dongteng, 6. Huangqin, 7. Jianghuang, 8. Ezhu, 9. Chuanxiong and 10. Gancao. To find the active ingredients of traditional Chinese medicine and related predicted targets. According to the TCMSP database user guide, the standard of oral bioavailability (OB) value > = 30%, drug similarity (DL) value > = 0.18 were set. Among them, Ren Dongteng was not found in the TCMSP database, and subsequently obtained from NERB (http://herb.ac.cn/) and ETCM (http://www.tcmip.cn/ETCM/index.php/Home/Index/) databases. Then the UniProt database (https://www.uniprot.org/) was applied to download the confirmed human genes, and the PERL software was used to convert and summarize the gene symbols of the full names of drug target genes.

### RA-associated genes search

Via GeneCards (https://www.genecards.org/), Online Mendelian Inheritance in Man (OMIM) (https://omim.org/), PharmGkb (https://www.pharmgkb.org/), therapeutic target database (TTD) (http://db.idrblab).net/ttd/), DrugBank (https://go.drugbank.com/), Rheumatoid Arthritis as the keyword was applied to search for disease-related genes. The R version 4.1.2 software was used to merge the disease-related genes obtained from the 5 databases, and the duplicated genes were deleted to draw a Venn diagram.

### Drug target-disease-related gene intersection

The R version 4.1.2 software was used to read drug target genes and disease genes, draw Venn diagrams, and obtain the intersection genes of drugs and diseases.

### Construction and analysis of network model

Using Cytoscape 3.9.0 and JAVA software, the interaction network diagram of “drug-ingredient-target” was constructed to obtain the relationship between active pharmaceutical ingredients and protein targets.

### Protein-protein interaction (PPI) network construction

The intersection of drug targets and disease-related genes was identified with the STRING database (https://cn.STRING-db.org/) and the database was employed to construct a PPI network model with the species set to “Homo sapiens” and a confidence score of > 0.9. A PPI network of protein interactions was constructed and ranked according to the degree of association between proteins.

The plug-in CytoNCA was applied to extract the nodes with higher scores in the PPI network, get the core genes, and found out the genes whose scores were greater than the median value. The filter conditions (BC: 56.003889815; CC: 0.208151383; DC: 7; EC:0.0356689; LAC:3; NC:3.7333333335) were set to get the gene list. The second network was screened with the filter condition (BC: 13.11746707; CC: 0.588235294; DC: 12; EC: 0.129669562; LAC: 6; NC: 7.120238095) to obtain key genes after two filters.

### Enrichment analysis of gene ontology (GO) function and Kyoto encyclopedia of genes and genomes (KEGG) pathway

The conversion of the drug-disease common target entrezID was completed by running the Bioconductor module package “org.Hs.eg.db” in the R software. The functional enrichment analysis of key target genes GO and KEGG was completed by “colorspace”, “STRINGi”, “ggplot2” and “DOSE”, “clusterProfiler”, and “enrichplot” modules in R software. Results were output in bar and bubble charts based on the transformed entrezID. The filter condition was P < 0.05, and the “pathview” module was run to draw a pathway map.

## Results

### Acquisition of potential active ingredients and targets of QED

The potential active ingredients and targets of QED were found in the TCMSP, NERB and ETCM databases as follows: Qianghuo has 16 main active ingredients and 96 targets; Duhuo has 9 main active ingredients and 94 targets; Qinjiao has 2 main active ingredients and targets 42; Yi Yiren has 10 main active ingredients and 49 targets; Ren Dongteng has 3 main active ingredients and 17 targets; Huangqin has 37 main active ingredients and 509 targets; Jianghuang has 3 main active ingredients and targets 42; Ezhu has 3 main active ingredients and 25 targets; Chuanxiong has 7 main active ingredients and 42 targets; Gancao has 93 main active ingredients and 1770 targets. There are 183 active ingredients in QED. After removing the potential active ingredients corresponding to duplicates and non-targets, there were 141 remaining active ingredients, and the number of corresponding targets was 271. The drug targets were annotated using UniProt database, and 237 target genes were obtained. The main medicinal ingredients of QED were showed in Table 1.

**Table 1:** Prediction of the main medicinal ingredients of QED

TCMSP MOLID	Active ingredient	OB (%)	DL	Ascribed Drugs
MOL000358	beta-sitosterol	36.91	0.75	Qianghuo/Duhuo/ Huangqin
MOL000449	Stigmasterol	43.83	0.76	Chuanxiong/Qinjiao
MOL002714	baicalein	33.52	0.21	Huangqin
MOL000173	wogonin	30.68	0.23	Huangqin
MOL000422	kaempferol	41.88	0.24	Gancao
MOL004792	nodakenin	57.12	0.69	Qianghuo/Duhuo
MOL001494	Mandenol	42	0.19	Chuanxiong/Yi Yiren
MOL001942	isoimperatorin	45.46	0.23	Qianghuo/Duhuo
MOL000953	cholesterol	37.87	0.68	Yi Yiren/Jianghuang
MOL000359	sitosterol	36.91	0.75	YiYiren/Jianghuang/Huangqin
MOL002135	Myricanone	40.6	0.51	Chuanxiong
MOL002140	Perlolyrine	65.95	0.27	Chuanxiong
MOL001942	isoimperatorin	45.46	0.23	Duhuo
MOL000296	hederagenin	36.91	0.75	Ezhu
MOL000525	Norwogonin	39.4	0.21	Huangqin
MOL000493	campesterol	37.58	0.71	Jianghuang
MOL006218	Methyl caffeate	30.68	0.06	Rendongteng
MOL000114	vanillic acid	35.47	0.04	Rendongteng
MOL001951	Bergaptin	41.73	0.42	Qianghuo
MOL001956	Cnidilin	32.69	0.28	Qianghuo

### Screening of rheumatoid arthritis-associated targets

**Figure 1:**
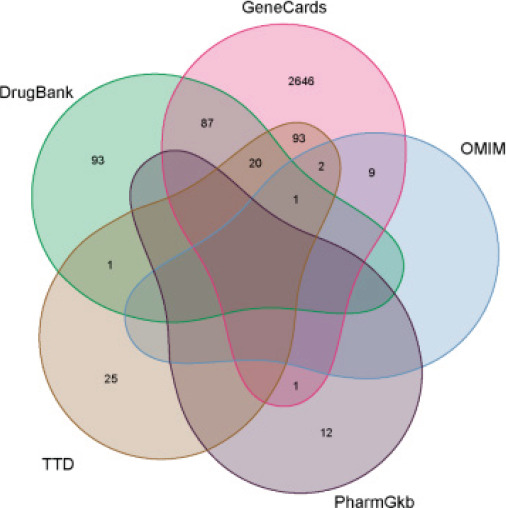
Summary of Rheumatoid Arthritis-associated targets

Retrieving disease target information Rheumatoid Arthritis-associated targets were obtained from the GeneCards, PharmGkb, OMIM, DrugBank, and TTD databases. The Venn diagram was obtained by taking the union of the disease genes obtained from the five databases. Among them, 2860 gene targets were obtained by GeneCards screening, 28 gene targets were obtained by OMIM screening, 14 gene targets were obtained by PharmGkb screening, 564 gene targets were obtained by DrugBank screening, and 149 gene targets were obtained by TTD screening. Duplicates were deleted, and 2990 disease targets related to rheumatoid arthritis were retained (Figure 1).

### Drug-disease common target intersection

**Figure 2:**
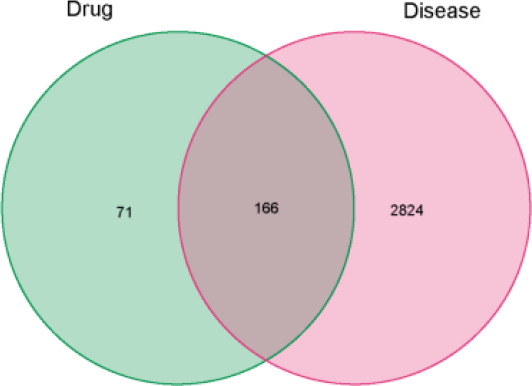
Visualization of the Chinese herbal compound ingredient-target network

A Venn diagram including the intersection between 237 drug targets and 2990 disease gene targets was drawn by R software, and a total of 166 drug-disease common targets were obtained, as shown in Figure 2.

### Construction of drug-component-target-disease interaction network

**Figure 3:**
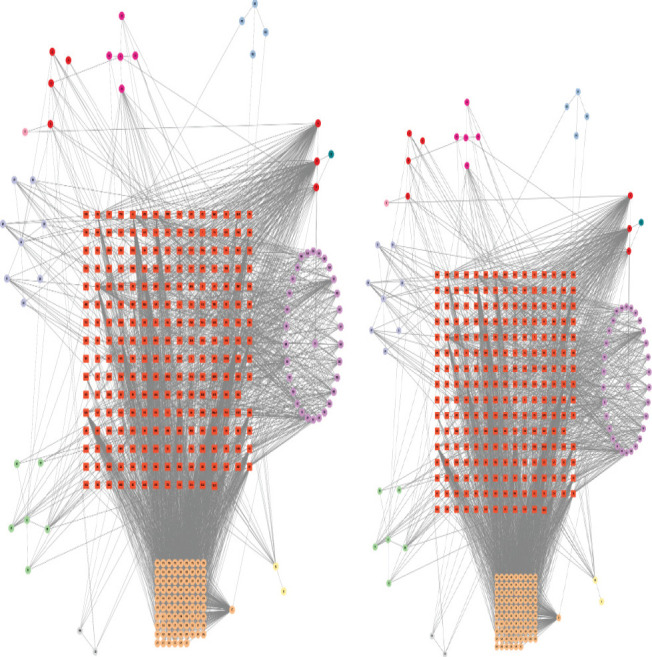
“Drug-ingredient-target” Network Diagram of QED. In this figure, the brick red represents the gene target, the lavender represents Qianghuo, the light green represents Duhuo, the pink-purple represents Huangqin, the dark green represents Qinjiao, the pink represents Jianghuang, the dark pink represents Chuanxiong, the blue represents Ren Dongteng, gray represents Yi Yiren, yellow represents Ezhu, and orange represents Gancao

The 166 drug-disease intersection genes, drug active ingredients and drug gene symbols collected in the above steps were entered into an Excel table, and the corresponding relationships and attributes were set, and then imported into Cytoscape 3.9.0 software to create a “drug-ingredient-target” map (see Figure 3). Among them, the intersection of active ingredients was shown in Table 2.

**Table 2:** The intersection of active ingredients

Name	Active Ingredients	herbs					
A1	MOL004792 nodakenin	Qianghuo	Duhuo				
B1	MOL001494 Mandenol	Chuanxiong	Yi Yiren				
A2	MOL001942 isoimperatorin	Qianghuo	Duhuo				
C1	MOL000953 cholesterol	Yi Yiren	Jianghuang				
D1	MOL000449 Stigmasterol	Yi Yiren	Jianghuang	Huangqin			
E1	MOL000358 beta-sitosterol	Qinjiao	Qianghuo	Duhuo	Huangqin		
F1	MOL000359 sitosterol	Chuanxiong	Qinjiao	Yi Yiren	Qianghuo	Gancao	Huangqin

### PPI network construction

**Figure 4:**
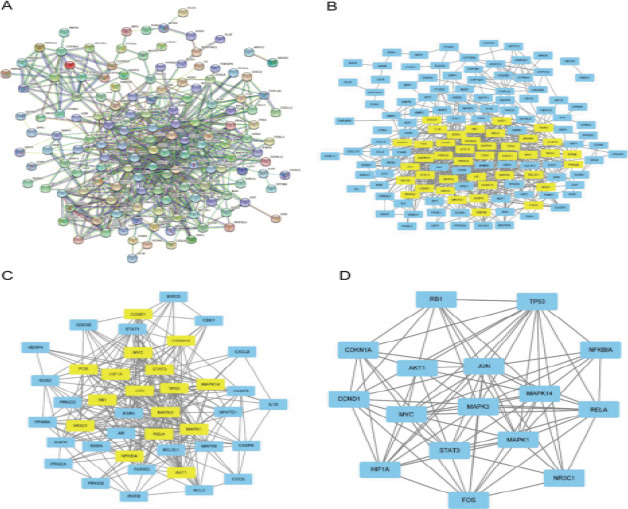
Interaction diagram of target proteins associated with QED

These 166 common targets were imported into the STRING PPI online software, the species item was checked as homo-sapiens, and the protein interaction relationship was obtained from the information in the database to construct a PPI network (see Figure 4). The score of each node was calculated by CytoNCA and preliminarily filtered, and the above CytoNCA steps were repeated to extract nodes with high scores, and finally a total of 16 core gene targets were obtained. PPI results were shown that the targets with higher frequency were: serine/threonine protein kinase (Akt), mitogen-activated protein kinase (MAPK), nuclear factor kappa B (NF-κB) inflammatory pathway, signal transduction and transcription activator 3 (STAT3), hypoxia-inducible factor 1 (HIF1), JUN gene, FOS gene, tumor protein 53 (TP53), etc. The information of the common targets was showed in Table 3.

**Table 3:** Protein introduction of key targets

Gene Name	Target	UniProt ID	Function	Structure
MAPK3	MAP kinase-activated protein kinase 3	Q16644	Catalytic activity/Activity regulation	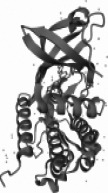
AKT1	RAC-alpha serine/threonine-protein kinase	P31749	Regulation of Apoptosis/Carbohydrate metabolism/Glucose metabolism/Glycogen biosynthesis/Glycogen metabolism	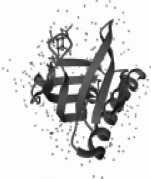
STAT3	Signal transducer and activator of transcription 3	P40763	Signal transducer and transcription activator	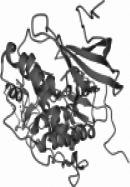
HIF1α	Hypoxia-inducible factor 1-alpha	Q16665	master transcriptional regulator of the adaptive response to hypoxia	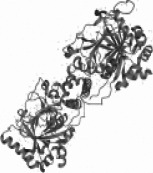
JUN	Transcription factor Jun	P05412	Transcription factor/Heterodimerizes with proteins of the FOS family to form an AP-1 transcription complex	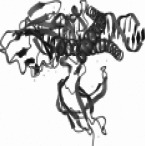
FOS	Protein c-Fos	P01100	Nuclear phosphoprotein which forms a tight but non-covalently linked complex with the JUN/AP-1 transcription factor	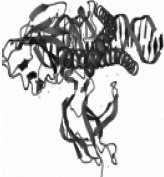
TP53	TP53-binding protein 1	Q12888	Double-strand break (DSB) repair protein involved in response to DNA damage	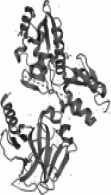
MYC	Myc protooncogene protein	P01106	Transcription factor/Activates the transcription of growth-related genes	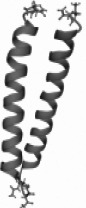
NFκB1	Nuclear factor NF-kappa-B p105 subunit	P19838	a pleiotropic transcription factor that are initiated by a vast array of stimuli related to many biological processes such as inflammation, immunity, differentiation, apoptosis.	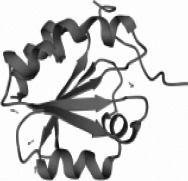
CCND1	G1/S-specific cyclin-D1	P24385	Regulatory component that phosphorylates and inhibits members of the retinoblastoma (RB) protein family and regulates the cell-cycle during G1/S transition	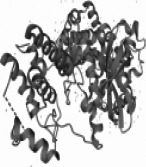

### GO enrichment and KEGG pathway analysis

**Figure 5:**
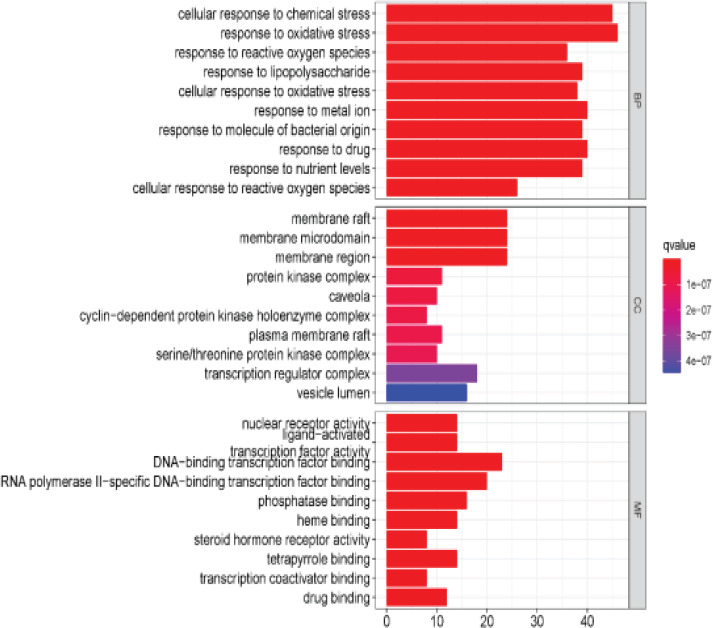
GO enrichment analysis

The 166 drug-disease common targets were analysed by R software, and the biological process, cellular component, molecular function of gene ontology (GO) was screened respectively. According to the number of enriched genes from large to small, the top 10 were selected for analysis (Figure 5). GO results showed that 2229 genes were enriched in biological process, accounting for 88.5%, which were the main influencing factors. The results mainly involved chemical stress, oxidative stress, reactive oxygen species, lipopolysaccharide, metal ions, bacteria source molecules, cellular responses at nutrient levels, etc. There were 78 genes enriched in cell components, accounting for 3%, mainly involving membrane rafts, membrane microdomains, membrane domains, protein kinase complexes, etc. There were 212 genes enriched in molecular function, accounting for 8.5%, mainly involving nuclear receptors, ligand/transcription factor activities, DNA-binding transcription factors, RNA polymerase II-specific DNA-binding transcription factors, protein phosphorylation, etc.

**Figure 6:**
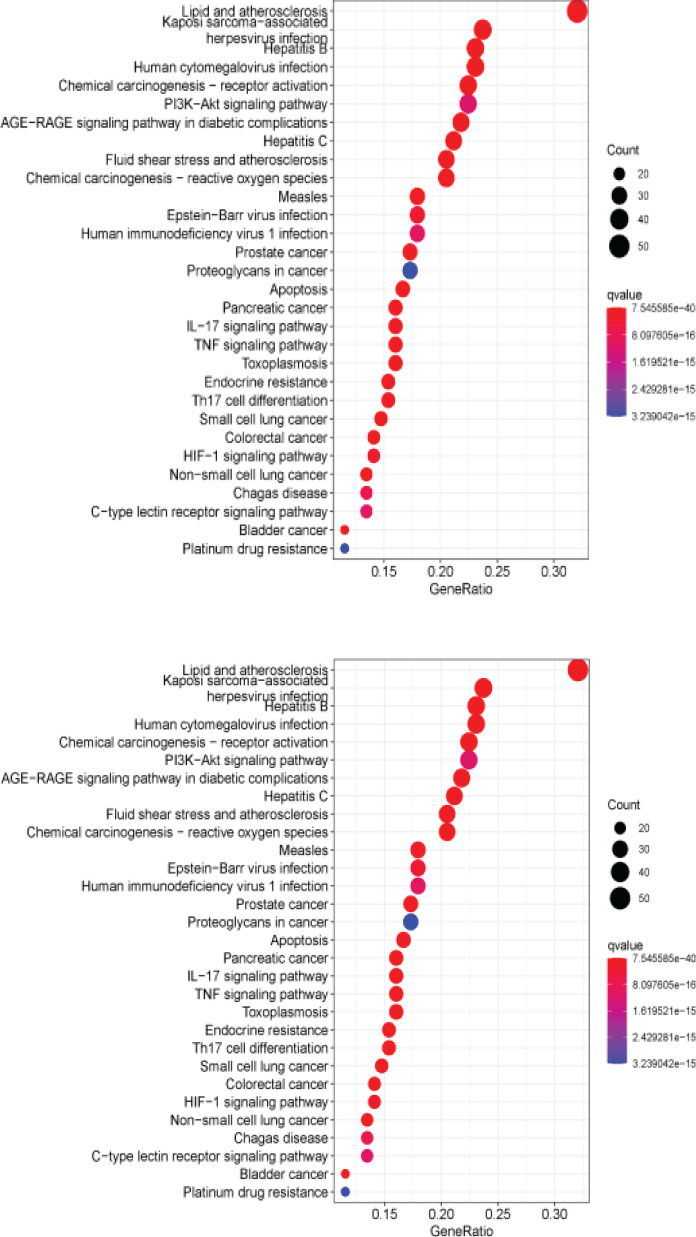
Top 30 of KEGG pathway enrichment analysis by QED

After running the 166 common targets through R software, 178 KEGG pathways were obtained. The top 30 functionally enriched KEGG bubble map results are shown in Figure 6. The abscissa in the figure represents the number of enrichments, and the ordinate represents q value. The smaller the q value, the redder the color, and the larger the q value, the bluer the color. The results of KEGG pathway enrichment analysis showed that common targets were mainly enriched in lipid and atherosclerosis, cancer pathway, phosphatidylinositol-3-kinase/protein kinase B (PI3K/Akt) signaling pathway, advanced glycosylation end products/receptor of AGEs (AGE-RAGE) pathway, interleukin 17 (IL-17), tumor necrosis factor (TNF) and other inflammatory signaling pathways and HIF-1 signal pathway.

## Discussion

In this study, 141 major active components, 237 potential targets and 166 overlapping targets of QHD with RA were identified, involving 2229 biological processes, 78 cellular components, 212 molecular functions and 178 KEGG-related signaling pathways. β-sitosterol, stigmasterol, baicalin and kaempferol were selected as the main active components of QHD. By regulating key targets such as Akt, MAPK, NF-κB, STAT3, HIF1α, JUN, FOS, TP53, and Multiple signaling pathway such as PI3K-Akt signaling pathway, AGE-RAGE signaling pathway, IL-17, TNF signaling pathway and HIF-1 signaling pathway plays a therapeutic role in RA.

In TCM, the etiology of RA is the deficiency of the body's righteous qi combined with the pathogenesis of wind, cold, dampness, heat and other pathogens that lead to the occurrence of the disease^5^,^12^. According to preliminary statistics, damp-heat and cold-damp impeding syndrome accounted for 81.5% of all RA patients admitted to our department. Most of these patients came from the hot and humid Chongqing region. Among them, the acute active phase of RA was mostly of damp-heat impeding syndrome, which belongs to the category of “Heat Bi Syndrome”^6^. Therefore, it is very important to clarify the mechanism about treating the damp-heat impeding syndrome of RA by QED.

The network analysis of drug active ingredients and targets in this study showed that active ingredients such as β-sitosterol, stigmasterol, baicalein, wogonin, and kaempferol in QED act on multiple targets. Modern research has shown that β-sitosterol has various biological activities such as lowering cholesterol, lowering blood sugar, antioxidant, anti-inflammatory, and antibacterial. In addition, it has also been confirmed that it can inhibit the expression of inflammatory factors by regulating the function of macrophages, to achieve the effect of the treatment of RA^13^,^14^. Stigmasterol can significantly inhibit the expression of pro-inflammatory mediators TNF-α, IL-6, IL-1β, inducible nitric oxide synthase (iNOS) and cyclooxygenase-2, and increase the anti-inflammatory cytokine IL-10 by downregulating the expression of NF-κB and MAPK pathways in joints expression^15^. Studies have shown that baicalein and wogonin can inhibit the expression of IL-1β, TNF-α and IL-18 by regulating the inflammasome NLRP3, NF-κB pathway, suppress the inflammatory response, and further achieve the effect of treating RA^16^,^17^. Kaempferol can inhibit the migration and invasion of fibroblast-like synovial cells in RA by blocking the activation of MAPK pathway, and attenuate the damage of RA^18^. Similarly, kaempferol inhibits cell proliferation and induces apoptosis in the treatment of RA, and relieves inflammation by inhibiting nuclear factor (NF-κB) and Akt/mTOR pathways^19^. Another study showed^20^ that Kaempferol acts as an antioxidant and anti-inflammatory agent by inhibiting nitric oxide synthase and cyclooxygenase-2 (COX-2) enzymes in RA.

The results of PPI network showed that: Akt, MAPK, NF-κB, STAT3, HIF1α, JUN, FOS, TP53 and other targets were the core targets of QED in the treatment of RA. The Akt plays an important role in cell survival and apoptosis, and regulates cell apoptosis in various ways^21^. The MAPK signaling pathway plays an important regulatory role in the process of cell proliferation, differentiation and apoptosis, and abnormal activation of MAPK often leads to the occurrence of RA^22^,^23^. The NF-κB signaling pathway is a bridge between cytokines and inflammatory responses, and RA is an inflammation-related disease whose occurrence and development are related to the excessive activation of NF-κB^24^. Activation of STAT3 in the process of RA can promote the expression of target gene matrix metalloproteinase, leading to cartilage lesions^25^. Inhibition of HIF1α expression can reduce angiogenesis in joint synovial tissue and reduce bone erosion in rheumatoid arthritis^26^. In addition, there are related reports that the occurrence and development of RA are often accompanied by mutations in JUN, FOS, and TP53 oncogenes^27^,^28^.

The GO enrichment results mainly involved: chemical stress, oxidative stress, reactive oxygen species, lipopolysaccharide cellular response, membrane rafts, membrane microdomains, membrane domains, nuclear receptors, ligand/transcription factor activities, DNA-binding transcription factors, etc. The KEGG results also showed that the therapeutic effect of QED on RA is mainly through: regulating lipid and atherosclerosis, cancer pathway, PI3K-Akt signaling pathway, AGE-RAGE pathway, IL-17, TNF and other inflammatory signaling pathways and HIF-1 signaling pathway. Recent studies have shown that RA is often complicated by cardiovascular disease, and dietary intervention using omega-3 polyunsaturated fatty acids has been shown to significantly improve RA symptoms, suggesting that the regulation of lipid metabolism may play a crucial role in the treatment of autoimmune diseases^29^. Studies have shown that PI3K/Akt acts on mammalian rapamycin target protein (mTOR), inhibits autophagy of fibroblast-like synovial cells, leads to the continuous proliferation of synovial cells, and aggravates the condition of RA^30^. In addition to this, the PI3K/Akt signaling pathway also affects the differentiation, generation and migration of osteoclasts, destroys bone and articular cartilage, and ultimately leads to joint deformities and aggravates the development of RA^31^.

The AGEs are toxic and irreversible compounds formed by a series of non-enzymatic reactions, which are produced by the active carbonyl groups of reducing sugars (such as glucose, fructose, glyoxal, etc.) and the free amino groups of proteins, fats and nucleic acids. The AGE-RAGE signaling pathway has been shown to mediate a variety of cellular damage processes^32^. In RA, the occurrence of oxidative stress in diseased cells increases the expression of AGE, leading to cytotoxicity and aggravating the condition of RA^33^. The IL-17 and TNF-α play a key role in inflammatory response and immune regulation, and the serum levels of RA patients are often accompanied by elevated expression of IL-17 and TNF-α^34^.

Research showed that^35^ TNF is a key cytokine that induces a wide range of intracellular signaling pathways, including apoptosis, inflammation, and immunity. Its expression in joint synovial fluid and synovial tissue of RA patients is significantly increased, which can affect synovial macrophages, synovial cells, chondrocytes and osteoclasts and further aggravate joint destruction.

In conclusion, the study explored the therapeutic effect of QED on RA and the corresponding molecular mechanism based on the network pharmacology method. The research results show that QED mainly treats RA by regulating inflammation-related signaling pathways, angiogenesis pathways, and reducing the expression of inflammatory factors. Though the study provides a theoretical basis for clinical treatment, the specific molecular mechanism still needs to be further studied and clarified.
